# Joint universal modular plasmids (JUMP): a flexible vector platform for synthetic biology

**DOI:** 10.1093/synbio/ysab003

**Published:** 2021-02-02

**Authors:** Marcos Valenzuela-Ortega, Christopher French

**Affiliations:** 1 Centre for Systems and Synthetic Biology, School of Biological Sciences, University of Edinburgh, Roger Land Building, Alexander Crum Brown Road, Edinburgh EH9 3FF, UK; 2 Zhejiang University-University of Edinburgh Joint Research Centre for Engineering Biology, International Campus, Zhejiang University, Haining, Zhejiang 314400, China

**Keywords:** DNA assembly, modular cloning, synthetic biology, SEVA, Golden Gate cloning

## Abstract

Generation of new DNA constructs is an essential process in modern life science and biotechnology. Modular cloning systems based on Golden Gate cloning, using Type IIS restriction endonucleases, allow assembly of complex multipart constructs from reusable basic DNA parts in a rapid, reliable and automation-friendly way. Many such toolkits are available, with varying degrees of compatibility, most of which are aimed at specific host organisms. Here, we present a vector design which allows simple vector modification by using modular cloning to assemble and add new functions in secondary sites flanking the main insertion site (used for conventional modular cloning). Assembly in all sites is compatible with the PhytoBricks standard, and vectors are compatible with the Standard European Vector Architecture (SEVA) as well as BioBricks. We demonstrate that this facilitates the construction of vectors with tailored functions and simplifies the workflow for generating libraries of constructs with common elements. We have made available a collection of vectors with 10 different microbial replication origins, varying in copy number and host range, and allowing chromosomal integration, as well as a selection of commonly used basic parts. This design expands the range of hosts which can be easily modified by modular cloning and acts as a toolkit which can be used to facilitate the generation of new toolkits with specific functions required for targeting further hosts.

## 1. Introduction

Synthetic biology is facilitated by DNA assembly systems which allow rapid generation of new constructs (1). Despite DNA synthesis becoming increasingly more affordable, the function of new DNA is hard to predict due to strong context dependency caused by interaction between recombinant genes and with host elements (2–4). Synthetic biology researchers have to reiterate the design–build–test cycle several times until the new system is optimized or tuned and, consequently, DNA assembly standards that are robust, automatable and accept reusable DNA parts are still necessary (5).

**Figure 1.**
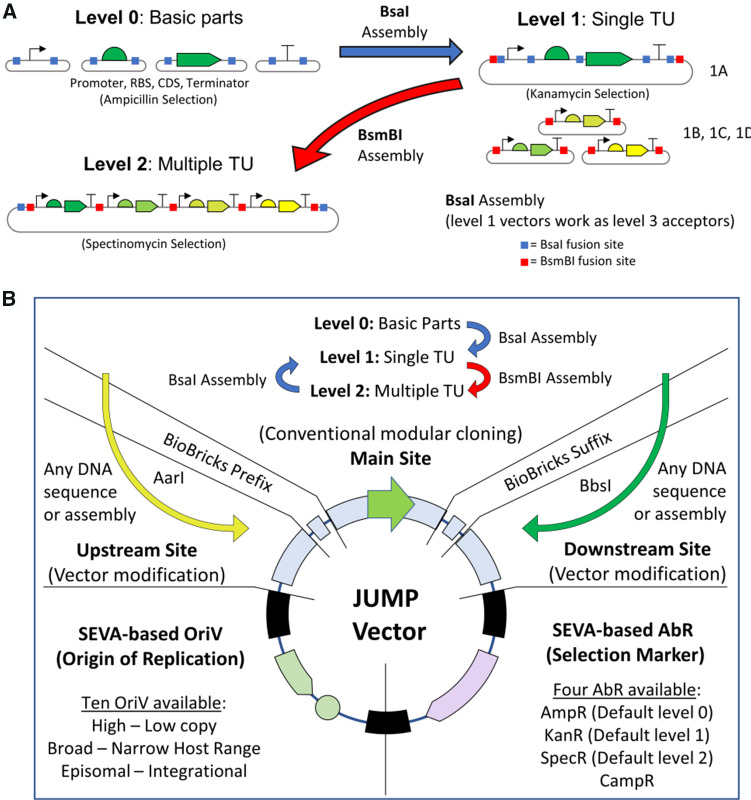
Modular cloning and general design of Joint Universal Modular Plasmids. (**A**) Basic parts are contained in level 0 vectors, which are assembled to form a single Transcription Unit (TU) in a level 1 vector (either 1A, 1B, 1C or 1D). To simplify cloning and screening, the destination vector contains a different selection marker than insert-donor plasmids and a reporter gene replaced by the assembled TU. Using a second type IIS restriction enzyme and selection marker, multiple level 1 assemblies (1A, 1B, 1C and 1D) can be combined in a level 2 vector (2A, 2B, 2C or 2D). In JUMP, level 1 plasmids can be used as level 3 assembly destination vectors. (**B**) The design of JUMP vectors combines modular cloning in the main site (as shown in A), compatibility with BioBricks and SEVA, and two orthogonal secondary modular cloning sites to introduce new features to any vector.

While Gibson assembly (6) and other overlap-based methodologies (as reviewed by Casini *et al.* (7)) are very efficient in assembling multiple DNA parts, standards based on Golden Gate cloning are the best fit for automation and part reusability (7). In Golden Gate cloning (8), DNA parts are flanked by sites recognized by type IIS restriction enzymes, which cut outside the recognized site, leaving user-specified overhangs (or fusion sites). Parts are ligated in an ordered manner, and restriction sites are removed during assembly, with assemblies of up to 24 parts (9). Different hierarchical standards based on Golden Gate have been published (10–19), often generically referred to as modular cloning or ‘MoClo’ after one of the first systems (11). In these standards, multiple level 0 parts (‘basic’ parts such as promoter elements, ribosome binding sites, coding sequences, N- or C-terminal tags, transcription termination sequences, etc.) are initially assembled in a specific order specified by the DNA overhangs generated by cleavage by a Type IIS restriction endonuclease and are inserted into level 1 acceptor vectors, to generate level 1 assemblies such as a transcription unit (TU) with promoter, ribosome binding site, open reading frame and transcription termination sequence. Typically, multiple level 1 assemblies can then be joined to form level 2 assemblies, level 2 assemblies to form level 3 assemblies, and so on (Figure 1A). Prior to such use, DNA parts need to be ‘domesticated’, a process that removes internal ‘forbidden’ restriction sites recognized by the enzymes used in the assembly process and adds flanking restriction sites which, on cleavage, will generate the appropriate overhangs for assembly, normally also introducing the part into a plasmid for amplification and distribution. After a part has been domesticated, Golden Gate-based standards are PCR-independent, allow re-use of parts in different assemblies and allow rapid generation of large and complex constructs. This is a big advantage over overlap-based assembly systems, as PCR reactions can fail and are prone to introduce mutations and, consequently, impose the need for more extensive quality control of the constructs generated.

The different Golden Gate-based standards have a similar hierarchical design but differ in the restriction enzymes used, fusion sites at different junctions, number of vectors needed, use of linkers and elements present in the fixed parts of the vectors, such as replication origins, selection markers and other elements required for particular hosts (Figure 1A).

To allow compatibility of basic parts, multiple toolkits have adopted the PhytoBrick (20) common syntax that dictates that BsaI must flank level 0 parts with specific fusion sites (Supplementary Figure S2). The PhytoBrick Standard was originally agreed among the plant synthetic biology community but is also used in standards compatible with bacteria (15, 16, 18).

For specific applications, some Golden Gate-based toolkits include special features in their vectors. For example, plant toolkits include left and right border sequences flanking the cloning site to allow *Agrobacterium*-mediated plant transformation (10, 11, 15, 16, 19), and some kits include unique flanking nucleotide sequences to allow the combination of multiple assemblies via Gibson assembly (16). Alternatively, some toolkits extend the possible uses by allowing the addition of features to vectors as additional parts, such as selection markers for target hosts or homology arms for chromosomal integration (18, 19). This strategy, however, increases the complexity of the assembly and might require multiple assembly steps. A similar situation occurs when multiple (or libraries of) constructs are to be generated which include common parts as well as variable parts. Examples include when inducible promoters are to be tested in the presence of a common transcription factor (or vice versa), or a library of guide RNAs with constant Cas9 or dCas9, or a library comprising a metabolic pathway with some genes variable and others constant.

An interesting feature partially addressing this is found in the EcoFlex toolkit (12). A subset of vectors includes a ‘secondary module’ to simplify pathway optimization. Multiple components (level 1 assemblies) can be introduced in the secondary module (which uses a different Type IIS restriction enzyme to that used for assembly in the main site), essentially modifying the vector, followed by a further assembly in the main site. Moore *et al.* (12) showed that secondary sites increase assembly efficiency by decreasing the number of parts required in each assembly reaction, which is highly desirable when generating libraries of assemblies. While the potential of secondary sites was demonstrated, it was limited by its design in EcoFlex plasmids, being restricted to special level 2 vectors that could only receive 2 or 3 level 1 assemblies in the main site, and these had to be pre-assembled into a level 2 vector before sub-cloning them into the secondary site. Similarly, the original MoClo standard (11) also allowed serial addition of level 2 assemblies into a single vector; this required a large library of linker parts and was restricted to level 2 acceptor vectors.

**Figure 2.**
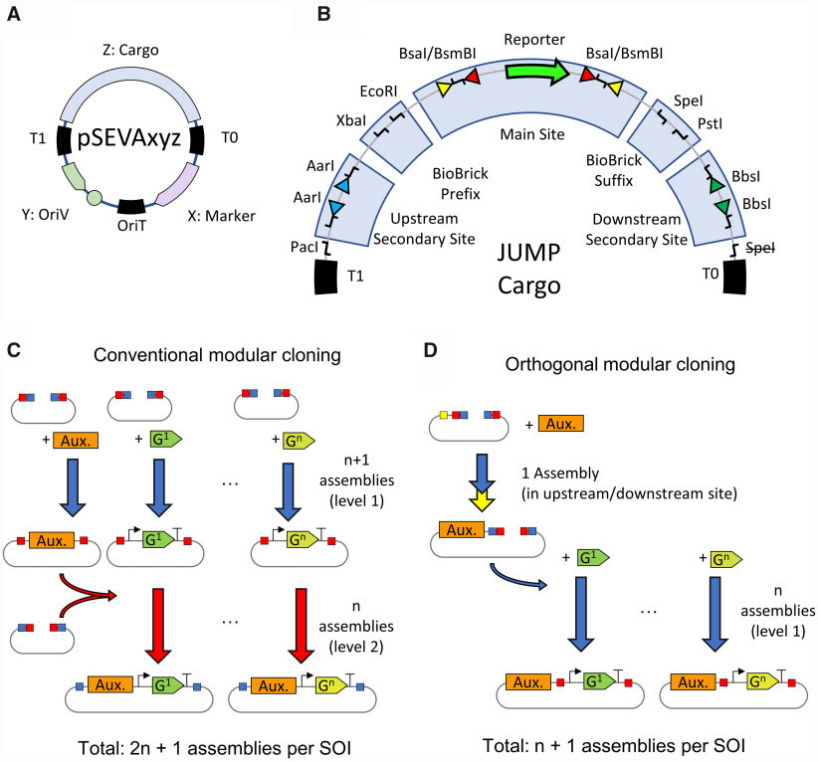
JUMP design and secondary sites. (**A**) In Standard European Vector Architecture (SEVA) plasmids, three common short DNA sequences (black) flank three variable regions (colored). Variable regions are the OriV (origin of replication), AbR (antibiotic selection marker) and ‘cargo’ (any expression cassette). The invariable regions are two transcription terminators flanking the cargo (T1 and T0) and origin of conjugation (oriT). Invariable regions also contain rare cutting sites, forbidden in the sequence of variable regions. (**B**) JUMP is designed as a special cargo of SEVA vectors to allow compatibility with future OriV's and AbR's of the collection. The cargo contains the upstream modular site (with outward-facing AarI sites); BioBrick prefix (XbaI, EcoRI); main modular site (a screening reporter gene flanked by outwards-facing BsaI and inwards-facing BsmBI sites for level 1, and vice versa for level 2); BioBrick suffix (SpeI, PstI); and downstream modular site (with outwards-facing BbsI sites). SEVA's canonical SpeI site was removed to allow BioBrick compatibility. (**C**) Building constructs to test similar genes (G^1^ to G^n^) as sequences of interest (SOI) that depend on common auxiliary factors (Aux.) with conventional modular cloning might require multiple assembly steps per SOI: the SOI is first assembled by itself and then combined with the auxiliary elements. (**D**) Introduction of the auxiliary factors in vector chassis using orthogonal use of secondary sites reduces number of assembly steps to combine the SOI with the auxiliary factor. Squares indicate restriction sites for BsaI (blue), BsmBI (red), AarI (yellow) and BbsI (green).

Here, we present ‘Joint Universal Modular Plasmids’ (JUMP), a vector standard designed to improve the flexibility of current modular cloning systems (Figure 1B). JUMP vectors allow streamlined assembly and combine compatibility with PhytoBricks (20) and BioBricks (21), with backbones based on the Standard European Vector Architecture (SEVA) (22–24). The design of SEVA allows the simple exchange of different elements of the vector (origins of replication (OriV), antibiotic selection markers (AbR) and cargo), and the SEVA repository comprises an ever-growing collection of standard vectors with different but compatible OriV and AbR (Figure 2A). Moreover, all JUMP vectors include two orthogonal secondary sites, one upstream of the main insertion site and the other downstream, that can directly receive inserts from any modular level (Figure 2B). Thus, researchers can easily modify the vector chassis to reduce the steps needed for iterative assemblies and to give vectors new features (Figure 2C and D). While no toolkit can be suitable for all purposes, JUMP facilitates the modification of vectors to generate new toolkits which can be used for a wide variety of purposes.

## 2. Methodology

### 2.1 Strains and media

*Escherichia coli* JM109 (New England Biolabs Ltd (NEB)) was used for transformations with assemblies. Selection and proliferation of strains were done with LB medium (10 g/l tryptone, 5 g/l yeast extract and 10 g/l NaCl) with the corresponding antibiotic depending on the plasmid (100 µg/l carbenicillin for level 0 vectors, 50 µg/l kanamycin for level 1 vectors and spectinomycin at 50 µg/l for level 2 vectors). Vectors with chloramphenicol resistance were selected at 18 µg/ml. Homemade chemically competent cells were prepared based on the protocol of Chung *et al.* (25).

Conjugation of plasmids was performed as described by de Lorenzo and Timmis (26): 0.1 ml of overnight LB cultures of the donor, recipient and helper strain (*E. coli* with plasmid RK600 coding *tra* genes, kindly provided by Dr Aitor de las Heras), were mixed with 5 ml of fresh LB and incubated at 30°C for 5 h, then 0.1 ml were streaked in selective medium (M9 medium with citrate as carbon source for *Pseudomonas putida*).

### 2.2 Vector construction

SEVA vectors were kindly provided by Prof. Victor de Lorenzo and his research group (22–24). JUMP vectors were generated by modifying the replication origins (OriV) and antibiotic selection markers (AbR) of SEVA vectors, and creating a new ‘cargo’ region as shown in Supplementary Figure S1. ‘Forbidden’ restriction sites (i.e. those that might be used in assembly reactions; specifically, BsaI, BsmBI, BbsI, AarI and BtgZI) were removed from SEVA components by site-directed mutagenesis as shown in Supplementary Tables S1 and S2. All vectors created are listed in Supplementary Table S3 and are available through AddGene (https://www.addgene.org/browse/article/28203402/) and their sequences are available in the Supplementary material.

### 2.3 Part domestication

Basic parts used here were domesticated by removing BsaI and BsmBI sites for compatibility with JUMP level 1 and 2 assemblies. We also routinely removed AarI, BbsI, BtgZ sites to allow assemblies using those enzymes for compatibility with other Golden Gate-based systems. We generated short parts (up to 120 bp) with annealed oligonucleotides and longer parts either by PCR or by DNA synthesis (Integrated DNA Technologies). To domesticate parts via PCR and remove internal forbidden sites, we followed a protocol based on Sarrion-Perdigones *et al.* (10), successfully domesticating parts with up to five internal forbidden sites (Supplementary Figure S3B). Parts are PCR-amplified between ends and internal forbidden sites as different sub-parts, which are assembled in the part-acceptor vector using BsmBI. The parts deposited in Addgene (including promoters, ribosome binding sites, open reading frames, transcription terminators and others) are listed in Supplementary Table S4, and their sequences are available in the Supplementary material.

We domesticated parts in the universal part-acceptor vector pJUMP18-Uac, which generates basic parts with any fusion site with overlapping BsaI and BtgZI sites. DNA fragments flanked by overlapping BsaI and BsmBI sites can be used for both part domestication into pJUMP18-Uac and directly for level 1 assembly (Supplementary Figure S3A). Promoters and terminators can be domesticated into the universal part acceptor or the specialized acceptors pJUMP19-Pac (*p*romoter *ac*ceptor) and pJUMP19-Tac (*t*erminator *ac*ceptor), which allow part characterization in *E. coli* as described in Section 3.

Basic (level 0) parts in the JUMP toolkit follow the PhytoBrick standard (18) as shown in Supplementary Figure S2. Most parts in the toolkit were built from BioBrick parts (21) or amplified from the genome of *E. coli* JM109. Terminators, as well as RBS PET, were adapted from the EcoFlex toolkit (12). The sequence of counter-selection coding sequence PheS A294G was obtained from Meyer *et al.* (27), the Lambda Red coding sequences were obtained from pKD46 and cI^ts^ (temperature-sensitive cI repressor) was obtained from pCP20 (28, 29).

The correct sequence of all parts and modified vectors was confirmed by Sanger sequencing carried out by Edinburgh Genomics.

### 2.4 Assembly conditions

Assembly conditions in the main site were as described for CIDAR-MoClo (13) and are explained in detail in Supplementary Methodology. Briefly, 20 fmol of all parts and destination vector were cyclically digested—with BsaI (NEB) at 37°C or BsmBI (NEB) at 42°C—and ligated with T4 DNA ligase (NEB) at 16°C (hereafter named BsaI assembly and BsmBI assembly, respectively). Double-stranded oligonucleotide linker ‘dummy parts’ were used to replace parts and reduce the number of inserts in some assemblies.

Assemblies into the upstream or downstream secondary sites of JUMP vectors were performed in two steps, without purification. In the first step, equimolar amounts of the inserts are assembled without destination vector as in conventional modular cloning assemblies. Meanwhile, the destination vector is digested with AarI (Thermo Fisher Scientific Inc.) or BbsI-HF (NEB) and Shrimp Alkaline Phosphatase (NEB). In the second step, equimolar amounts of insert assembly and destination vector are ligated with freshly added T4 ligase enzyme and T4 ligase buffer (NEB) for one hour at 16°C.

One microliter of assembly reactions was used to transform *E. coli* and transformed colonies appearing in the presence of the antibiotic corresponding to the destination plasmid were screened by colony PCR and restriction digestion of plasmid DNA minipreps. Assembly into the main site (but not the upstream or downstream secondary sites) of JUMP vectors replaces an sfGFP cassette, with constitutive promoter J23100, which can be detected in colonies using a blue-light source such as ImagerTM Blue-Light Transilluminator (Invitrogen) with an amber filter unit. During the screening, we additionally checked for antibiotic resistance of transformed clones to ensure that only the destination vector had been transformed, due to the serendipitous finding that insert-carrying plasmids co-transform simultaneously with the destination plasmid even without the presence of their specific selecting antibiotic. We found that this also occurred using other toolkits, and co-transformed colonies, containing two different plasmids, were sometimes more than 10% of the total (data not shown).

Detailed protocols for JUMP plasmids have been published separately (30). SnapGene software (GSL Biotech LLC) was used in the design processes of vector construction, part domestication and assembly planning. Open access design-automation tools, such as those made available by the Edinburgh Genome Foundry (https://cuba.genomefoundry.org/) can also be used to plan assemblies and quality-control checks.

### 2.5 Fluorescence measurements

Expression of sfGFP was measured to determine gene expression from different JUMP vectors. Absorbance and fluorescence were measured in a plate reader (FLUOstar Omega Microplate Reader, BMG Labtech). Overnight cultures were diluted 5-fold, and green fluorescence was measured in triplicate, with excitation and emission wavelengths of 485 and 520 nm, respectively, and was normalized by optical density measured at 600 nm. To characterize promoters and terminators, eGFP expression was measured as explained above, and relative fluorescence was normalized to that of the J23100 promoter (31). The characterization of constructs with mCherry as reporter was done by fluorescence with excitation and emission wavelengths of 584 and 610 nm.

## 3. Results and discussion

### 3.1 Design and construction of JUMP vector backbones

The philosophy of JUMP was to generate a new Golden Gate-based assembly system which would build on the advantages offered by various different systems currently available while also allowing direct use in multiple species of bacteria, as well as easy modification of vectors for new hosts and new applications, offering maximum flexibility and applicability. To this end, it was decided: that vectors would be based on the SEVA system, which offers wide flexibility and applicability to different bacterial species; that level 0 parts should be based on the PhytoBrick standard, already accepted by iGEM and used in multiple other Golden Gate-based kits; that BioBrick compatibility would be included, for backwards compatibility with constructs in the Registry of Standard Biological Parts; and that the main cloning site in all level 1 and level 2 vectors would be flanked by upstream and downstream secondary sites, which would also allow both level 1 and level 2 assembly directly into these sites. The design is summarized in Figures 1 and 2.

Construction of JUMP vectors is explained in detail in Supplementary Figure S1. Restriction sites relevant to cloning in JUMP and other PhytoBrick-compatible systems (BsaI, BsmBI, BbsI, AarI and BtgZI) were removed from SEVA components as shown in Supplementary Tables S1 and S2. The JUMP ‘cargo’ (Figure 2B), consisting of upstream cloning site flanked by AarI sites, BioBrick prefix, main modular cloning site consisting of marker gene sfGFP flanked by BsaI and BsmBI sites, BioBrick suffix and downstream cloning site flanked by BbsI sites, was then introduced. Level 1 (with main modules 1A, 1B, 1C and 1D) and level 2 (with main modules 2A, 2B, 2C and 2D) vectors were built as a ‘core set’ using SEVA's medium copy-number origin of replication #9 (pBRR322/ROP), and alternative vectors with nine different replication origins were built with the main module 1A and 2A as shown in Supplementary Table S3. This architecture allows researchers to build intermediate assemblies with the core set and then perform the final assembly (either level 1 or level 2) with the OriV of choice. An example of a level 2 assembly appears in the Supplementary Figure S4. The default antibiotic markers are ampicillin/carbenicillin for level 0, kanamycin for level 1 and spectinomycin/streptomycin for level 2. Additional alternative vectors have been included in the toolkit with a different marker gene for screening (*lacZ’α* instead of sfGFP), and antibiotic marker (a broad-host range chloramphenicol resistance gene adapted from the shuttle vector pSEVA3b61, which we named AbR #3 in JUMP vectors for the sake of simplicity) (32). Additionally, we included two pairs of vectors that replace the vectors 1D (with 1D' and 1E) and 2D (with 2D' and 2E), which give the option of combining five inserts rather than the standard 4 in level 2 and level 3 assemblies. To replace the main site, the flanking BioBrick sites were used.

**Figure 3.**
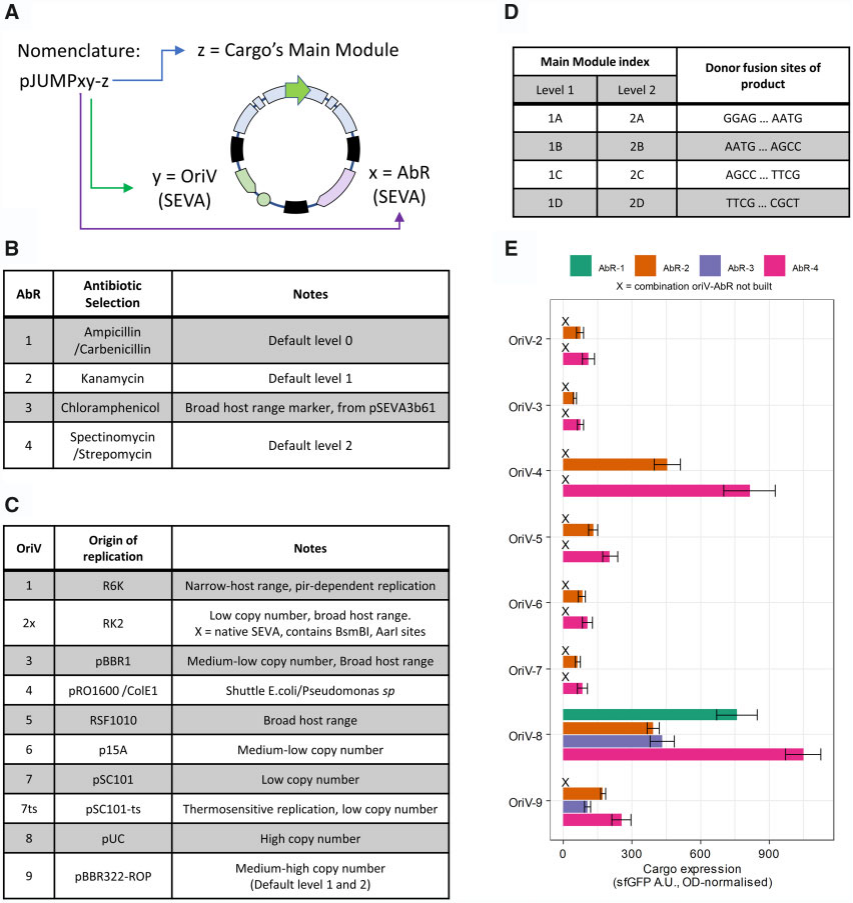
JUMP nomenclature and backbones. (**A**) JUMP follows SEVA nomenclature for origins of replication (OriV) and selection marker (AbR), while cargo nomenclature is replaced by JUMP's main site module index. (**B**) Antibiotic selection markers used in JUMP vectors. (**C**) Origins of replication in JUMP vectors. (**D**) Default main site modules and their donor fusion sites. (**E**) OD-normalized fluorescence of sfGFP cloning reporter in available combinations of OriV-AbR in the distributed JUMP toolkit. Error bars indicate standard deviation, *n* = 3.

The nomenclature of JUMP vectors (Figure 3A) is conservative with SEVA's (22–24) to facilitate the combination of new SEVA OriV and AbR with JUMP vectors. Of a vector ‘pJUMPxy-z’, x and y indicate the selection marker and origin of replication as indicated by SEVA rules, with the difference of relevant restriction sites being removed in the JUMP version (see Figure 3B and C). The characteristics of the main module sites—level and position—are indicated by the index z (Figure 3D). For example, vector pJUMP29-1A(sfGFP) has AbR #2 (Kanamycin), OriV #9 (pBBR322/ROP) and main site module level 1 that would take position A in a level 2 assembly. Note that JUMP vectors are not SEVA vectors, and should be considered as SEVA ‘siblings’, as they do not strictly follow the SEVA rules, due to the SEVA native SpeI site being removed to make JUMP vectors compatible with BioBricks.

To allow compatibility with the widely used PhytoBrick level 0 parts, all acceptor fusion sites are constant (GGAG and CGCT) in all main and secondary sites. In the main sites, hierarchical conventional modular cloning works similarly as in current standards (depicted in Figure 1A). PhytoBrick level 0 parts are assembled in any JUMP level 1 vector (of type 1A, 1B, 1C or 1D) using BsaI (indicated by the removal of the marker gene); four level 1 products (assembled in vectors of type 1A, 1B, 1C and 1D) can be combined in any level 2 vector using BsmBI. As with Mobius (15) and Loop assembly (16, 18), JUMP level 1 vectors can be used as level 3 assembly destinations for four level 2 products (assembled in vectors of type 2A, 2B, 2C and 2D) using BsaI. In addition to the compatibility with other toolkits, the use of constant acceptor sites offers the possibility to introduce sequences from any assembly level into either secondary site in any JUMP vector.

### 3.2 Use of different JUMP vectors

Most Golden Gate-based toolkits only offer vectors with one origin of replication. Two recent toolkits offer multiple microbial OriV; the uLoop kit (18) offers four OriV with different copy number, and the MK toolkit (19) offers four bacterial broad-host range OriV as well as two for yeast. A recent publication (33), offers four OriV in SEVA-derived plasmids named ‘SEVA 3.1’ that allow Golden Gate cloning but requires PCR-amplification of parts and vectors for each assembly, which is avoided by other Golden Gate-based assembly systems. The JUMP toolkit includes 10 OriV, including broad-host range and variable copy-number OriV as well as conditional vectors to allow chromosomal integration. Four replicative OriV allow its use with other hosts beyond *E. coli*, the *Pseudomonas* shuttle OriV #4 (pRO1600/ColE1) and three broad-host range vectors: origins #2x (RK2), #3 (pBBR1) and 5# (RSF1010). RK2 and pBBR1 are known to be replicative in a wide number of gram-negative bacteria, and the hyper-promiscuous RSF1010 has been shown to replicate in some gram-positive bacteria and yeast species (34). We confirmed that JUMP vectors can be trans-conjugated from *E. coli* to *P. putida* KT2440 (data not shown), using a tri-parental conjugation (26, 35). We successfully conjugated *P. putida* with level 1 vectors with OriV #2, #3 and #5. Unexpectedly, the *Pseudomonas* shuttle OriV #4 did not yield any conjugants in our tests, although it contains no changes in the sequence from the original SEVA oriV that has previously been introduced into *P. putida* (36). Direct transformation of *P. putida* by electroporation was not tested.

The availability of multiple vector backbones allows optimization of the expression of the genetic construct. This is important as the characteristics of the vector strongly affect the expression of the gene it carries through context dependency (the effect of interactions of biological host complex environment and the function of the recombinant gene of interest) (12, 13, 17, 37). Kim *et al.* (37) showed the relevance of ‘tuning’ the vector chassis by testing different constructs with multiple combinations of OriV and AbR's. While the highest copy-number vectors were found to impose a deleterious burden on host cells, other OriV's showed different expression levels depending on the AbR they were paired with. The JUMP toolkit allows researchers to choose among 10 different origins of replication and four antibiotic selection markers (Figure 3B and C). To show the effect of the vector chassis, we measured the expression of the constitutive sfGFP reporter, which is driven by promoter J23100 (31), in all empty vectors in the toolkit (Figure 3E). As expected, we found strong differences between different OriV and AbR. Expression was higher with the high-copy OriV #4 and #8, followed by the medium-copy #9, and the remaining OriV showed lower expression. The antibiotic resistance marker used also showed an effect on expression. AbR #4, (spectinomycin resistance) showed higher reporter expression than AbR #2 (kanamycin resistance) with all OriV. The fact that the difference increased with the copy number of the OriV suggests that this was caused by the expression of the resistance gene rather than the antibiotic itself.

The actual copy number of the vector was not quantified as it is known to vary depending on host strain, among other factors (38). These results were overall similar to those showed by the copy-number study of Jahn *et al.* (38), and the small differences (OriV #2 and #5 displaying slightly higher expression here) could be due to a different strain and different cargo being used. Nevertheless, these results show the value of testing different vectors if the expression of the assembled construct requires tuning for optimization.

The JUMP toolkit also offers conditional OriV's to allow chromosomal integration. The SEVA OriV #1 (R6K) depends on the *pir* gene, not present in most *E. coli* strains. We inserted the vector pJUMP21-1A in *E. coli* JM109 (*pir*-) by introducing a sequence homologous to the 2 kb upstream of *ldhA* in the upstream secondary site, obtaining kanamycin-resistant colonies in the *pir- E. coli* strain JM109 upon transformation. No transformants were obtained when the homology region was not included. Additionally, we built a thermosensitive oriV (#7ts) by replacing the Rep101 protein of SEVA's oriV #7 (pSC101-based) with its thermosensitive counterpart from the plasmid pCP20 (28, 29). After confirming that replication was temperature dependent (Supplementary Figure S6), we introduced a counter-selection marker, *pheS* mutant A294G (27), in the downstream secondary site to allow curable chromosomal integration, which we demonstrated with a scarless transcriptional fusion of *gltA* in *E. coli* MG1655 to RBS and sfGFP parts from the toolkit (Supplementary Figure S7). The presence of this cassette, correctly inserted at the *gltA* locus, was confirmed by PCR.

### 3.3. Use of upstream and downstream sites

While the main site enables modular cloning as with other Golden Gate-based systems, the advantage of JUMP resides in the presence of additional Golden Gate cloning sites in all vectors. The upstream and downstream secondary cloning sites use AarI and BbsI to introduce any sequence into the vectors, thus not disrupting the function of the main site of any vector (level 0, 1 or 2). By being able to introduce any sequence in either secondary site of any vector, researchers can simplify the cloning steps needed to test the gene, genetic device or sequence of interest (SOI) (Figure 2C and D). Many recombinant SOI require other common sequences to be present (auxiliary elements such as a transcription factor for inducible expression, a biosensor or reporter gene, Cas9/dCas9 for CRISPR, homology sequences for chromosomal integration, etc). JUMP allows such auxiliary elements to be introduced in a secondary site, thus reducing the number of assemblies when the auxiliary elements are common but multiple SOI will be assembled or require optimization.

**Figure 4.**
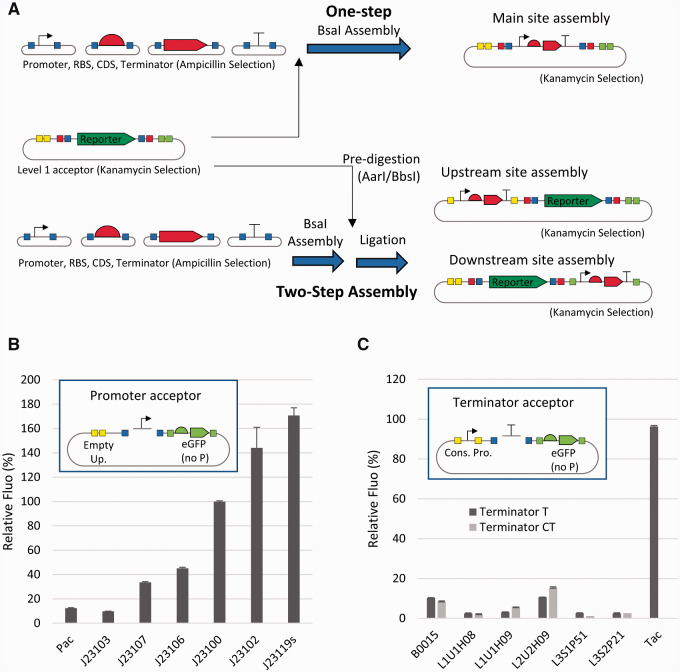
Use of secondary sites with two-step assembly. (**A**) Two-step assembly works by assembling inserts first and then ligating the assembly reaction with the destination vector. Squares indicate restriction sites for BsaI (blue), BsmBI (red), AarI (yellow) and BbsI (green). (**B**) Domestication and characterization of promoters using level-0 promoter acceptor. (**C**) Domestication and characterization of terminators using level-0 terminator acceptor. T and CT terminators differ in the 5' end of the part (Supplementary Figure S2), with CT terminators including a stop codon. In B and C, fluorescence was normalized to OD (600 nm) and is shown as % of that of the J23100 promoter. Error bars indicate standard deviation, *n* = 9 (biological and technical triplicate).

The potential of secondary sites resides in how easily they can be used. By having the same receiving fusion sites, they are capable of receiving inserts assembled in the main site via a classic modular cloning approach (in addition to conventional restriction–ligation cloning). A conventional Golden Gate assembly (one-pot and one-endonuclease) does not allow assembly of inserts into secondary sites because the inserts are assembled with BsaI/BsmBI and this would cut the destination vector in the main site. JUMP level 0 parts were originally designed with overlapping BsaI and BtgZI sites, to allow double-enzyme assemblies as shown by Sarrion-Perdigones *et al.* (10); however, one-pot one-step assembly with BtgZI digesting level 0 inserts and AarI or BbsI digesting the destination vector showed very poor efficiency, possibly due to BtgZI remaining attached to the DNA and interfering with the assembly (39). Therefore, we use an efficient two-step assembly approach (Figure 4A). In the first step, the basic parts were assembled in the same conditions as for classic level 1 assemblies, and the destination vector was digested separately with AarI or BbsI for the upstream or downstream site, respectively, and dephosphorylated. In the second step, the digested backbone is ligated with the insert assembly mix. This assembly method was tested by assembling an mCherry TU from level 0 parts in the secondary sites of a level 1 destination vector. The transformation of the assembly resulted in more than 90% correct (mCherry+) colonies (Supplementary Figure S5). We expect that this approach to modify vectors outside the conventional modular cloning site can easily be automated, as there is no need to perform any purification steps or PCR reactions to prepare inserts since inserts can come from the modular cloning assembly pipeline without further modification. Additionally, AarI and BbsI sites do not have to be removed from inserts, as the restriction sites used for assembly on the main site (BsaI/BsmBI) are used on the sequence introduced in secondary sites. The two-step assembly can be extended to any insert and destination vector provided that the external fusion sites of the insert match the receiving fusion site of the destination vector (conserved for all JUMP vectors donor and acceptor sites), and the destination vector is selected with a different antibiotic than the insert donor vectors. If the antibiotic selection of the donor and recipient vector match, clones with recipient vector should be distinguishable by expressing the cloning marker still present in the main site.

We used secondary sites to characterize promoters and terminators (listed in Supplementary Table S4 and available in Addgene) directly in the domestication (level 0) vector. We generated a level-0 promoter acceptor vector (pJUMP19-Pac) that reports the activity of the promoter by introducing eGFP without promoter in the downstream secondary site. During the domestication of promoters, the eGFP indicated the presence of the promoter and allowed characterization of the promoter strength (Figure 4B). We applied the same principle to domesticate and characterize terminators by introducing a constitutive promoter in the upstream secondary site, in such a way that the expression of the downstream eGFP would be disrupted by the presence of terminators in the main site (Figure 4C). Thus, part characterization can be done directly in the level 0 donor vector, rather than requiring level 1 or level 2 assemblies (12, 13).

**Figure 5.**
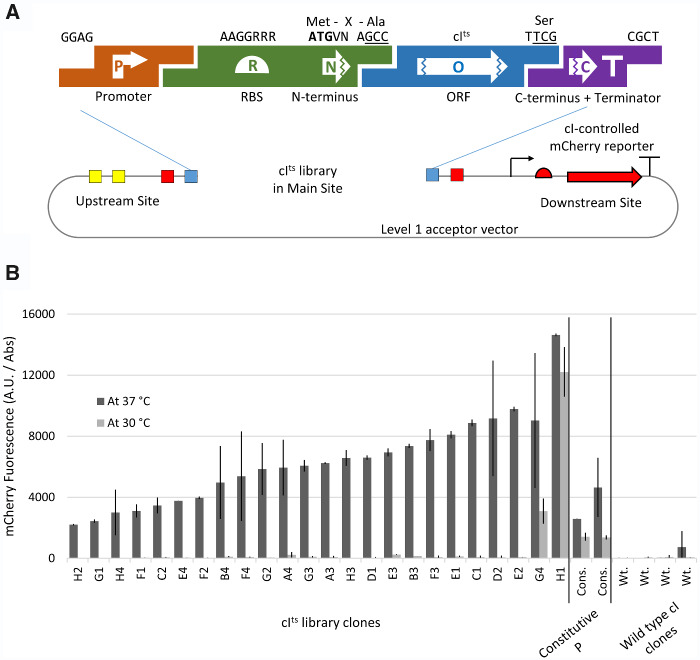
Use of secondary sites to screen variants of a transcription factor gene. (**A**) The parts used for the construction of the cI^ts^ library were: a constitutive promoter, an RBS+N-terminus part, cI^ts^ CDS without N or C terminus, and C-terminus + Terminator. The RBS+N-terminus part was PCR-built from a degenerate oligonucleotide variable for the Shine–Dalgarno consensus and for the codon following the start ATG, thus giving the library diversity in translation initiation and half-life of the protein (40). (**B**) Colonies that were white at 30°C were tested for expression of the mCherry reporter at 37°C. As a control, we analyzed the fluorescence of two colonies from the library that were unrepressed at 30°C (clones G4 and H1), two colonies with a constitutive mCherry gene, and four colonies from an equivalent library assembled with the wild-type (thermostable) cI CDS part.

A second example shows how the screening of a combinatorial assembly can be simplified using the secondary site (Figure 5). We generated a library of TU expressing different levels of the lambda phage repressor cI^ts^, which we domesticated from pCP20 (28, 29). This was done to optimize this TU before using it to regulate other genes in a later level 2 assembly (not shown). To characterize the clones of the library, a reporter with the promoter controlled by cI must be present. We reduced the number of assembly steps needed to one per cI^ts^ by building the library in a level 1 vector where we had previously introduced the reporter gene in the upstream secondary site (Figure 5A). The clones from the library should vary in translation and stability of transcription factor, therefore we expected variability in the expression of the mCherry reporter at 30°C and 37°C, which we confirmed (Figure 5B).

It is important to note that virtually any sequence can be introduced in secondary sites. Some recent publications (13–15) have shown that new housekeeping elements normally present in the vector chassis (e.g. selection markers) can be introduced as assembly parts, thus increasing toolkit flexibility at the expense of assembly complexity. By using secondary sites, any new feature can be introduced into JUMP vectors without increasing the complexity of later assemblies using the main cloning site. Therefore, tailored plasmids can be built for any application: auxiliary elements to test the assembly in the main site, recombinase recognition sites, counter-selection markers, homology sequences for chromosomal integration, alternative origins of replication, etc.

### 3.4 Toolkit distribution

A collection of 96 vectors is available in Addgene (https://www.addgene.org/browse/article/28203402/). This collection includes a selection of PhytoBrick-compatible level 0 parts for synthetic biology applications in bacteria, a basic part Universal Acceptor, the level 1 and level 2 vector ‘core set’, and alternative vectors. We have removed relevant type IIS restriction sites from all these basic parts and vectors, with the exception of the OriV #2x, (unmodified SEVA broad-host range oriV #2/R2K, which is BsaI free but contains other forbidden sites). Addgene kindly sequenced all the deposited plasmids confirming the absence of unwanted mutations.

## 4. Conclusions

JUMP has been designed as a platform to allow Type IIS-RE-based assemblies to be used in multiple bacterial hosts as well as allowing easy modification of the vectors to suit the requirements of new hosts. The capabilities of Golden Gate-based assembly are combined with the vector flexibility of SEVA and multiple cloning sites. The toolkit generated allows any assembly level be done with 10 different origins of replication. The vectors range from low to high copy number, include integrative vectors and can be used with a very wide list of non-model microorganisms. Since they are based on SEVA, future OriV's of the collection and AbR's added to the collection can be easily incorporated into JUMP vectors, and researchers can easily create new origins of replication and markers for the toolkit or use JUMP vectors with any PhytoBrick-compatible part. We have demonstrated that the upstream and downstream secondary sites expand the paradigm of modular cloning to allow orthogonal modification of the vector chassis.

## SUPPLEMENTARY DATA

Supplementary Data are available at *SYNBIO* Online.

## Funding

The Biotechnology and Biological Sciences Research Council [BB/J01446X/1]. Funding for open access charge: University of Edinburgh. 

*Conflict of interest statement*. None declared.

## Supplementary Material

ysab003_Supplementary_Data
